# Hospital Letters

**Published:** 1895-08-17

**Authors:** 


### HOSPITAL LETTERS.

(By a Correspondent.)

An article in a late number of The Hospital (July 20th) ended with these words, "The lines system is well worthy of free discussion, and we hope our calling attention to it may at any rate attain this end."

The writer of the article is against hospital lines, and in theory he is probably right, though for practical working there is much to be said in favour of lines under certain restrictions.

A voluntary hospital is, of course, subject to the rules those who maintain it choose to make. If the subscribers decide to admit free those who are in a position to pay for themselves, or servants, or employés, for whom others are responsible, why should they not do so? We may argue that their money might be better spent, but who is entitled to complain or to say it is an abuse of charity? No doubt the ideal of charity is that the money should be given to help those who are not in a position to help themselves, without any *quid pro quo* other than that which the doing of the charitable act itself brings. Yet it may not be wise in practice to ignore the very common desire in human beings to get some more tangible return for what is given. Bazaars are the great example of this. A sum of money is wanted for the best of objects. Why not simply ask for the money? It would be such a saving of trouble all round if people would only hand up the cash. Those who have tried know the answer. And so a bazaar is started, and people who would not give shillings before now spend pounds buying things they do not want. What is this but an example of a deeply engrained feeling in human nature—the desire to have something to show for your money? Is it not wise to take notice of this feeling, and to give hospital lines in some form for money subscribed? And, after all, is it unfair that those who provide the money should have some privileges?

There is also to be considered the converse of the case put in the article in question. Doubtless some masters, if they get the chance, take advantage of lines to save their own pockets. But take the case of a master subscribing liberally year by year to a hospital and yet seldom or never using one of his lines. At last a servant falls ill and is sent to hospital. Is it not rather shabby to refuse the servant admission except on the same footing as any other paying patient, and to ignore years of generosity on the part of the master?

To enter into all the points for and against lines would be too long a story here. Perhaps, however, it might be of use to describe the system lately adopted by the managers of the Ayr County Hospital after careful consideration of the whole subject.

This hospital is supported almost entirely by voluntary subscriptions, and the management is in the hands of the subscribers. Over £3,000 a year has to be raised. For every guinea subscribed a letter of recommendation is issued available for twelve months from the date of issue. On this printed letter or "line" must be inserted the proposed patient's and also the subscriber's name and address, and the following particulars as to the patient: Age, employment, constant or temporary, employer, wages, married or single, number and ages of family, total weekly earnings of members of family, how long ill, and what sum per week applicant will pay towards cost of treatment. (This is to meet the case of those who are neither entitled to free treatment nor are able to pay the full cost.) Attention is directed (in red type) to the hospital being a charity, and subscribers are enjoined not to give lines to those who, in the words of the hospital constitution, are able to provide for themselves medical advice and attendance, or for whom any private person or public body is responsible, such persons being admissible only as paying patients. (Paying patients are only admitted when there are no free patients to fill the beds.) Accidents are admitted without lines. To meet cases such as have already been referred to it is provided that if subscribers send their own servants or employés as paying patients (which is the only footing on which they are admitted) the subscribers' own lines will be accepted in payment of the cost of treatment at the rate of one line for each guinea. This allows the master if he pleases to receive free treatment for his servant up to the amount of his annual subscription, but no further, while the master who takes a higher view of what true charity is need not claim the privilege.

This system appears to meet most of the objections which are urged against lines as well as the reasons urged in their favour. It is not here put forward as an absolutely perfect system, but as a thoughtful attempt to deal with an important and difficult point in hospital administration which may be of some use to those interested in the subject.

*** Much depends on the definition of the word "charity," and although we may admit that subscribers have the right to make what rules they like, we can hardly allow that people who "are in a position to pay for themselves" should be admitted free to an institution called a charity, even when provided with a ticket. Moreover, however great may be the desire of the average Briton to obtain a *quid pro quo*, it seems an obvious abuse of the charity to which he subscribes, when, by the use of a "letter," a subsriber obtains treatment worth many times the money value of his subscription for an employé for whose treatment he is responsible. Certainly the plan described by our correspondent as in operation at Ayr is good and practical, especially in this respect, that, if the patients admitted are such that they ought to pay, the subscriber's letter only franks them to the extent of its own money value. The true solution of many difficulties in hospital management lies in the frank acceptance of the principle that those who can pay will have to pay, and nothing stands in the way of this so much as the fact that by a little manœuvring people who can well afford to pay for themselves can obtain "hospital letters" admitting them free to all the benefits of hospital treatment.—Ed. *T. H.*

